# Solid-Phase Partitioning and Leaching Behavior of Pb and Zn from Playground Soils in Kabwe, Zambia

**DOI:** 10.3390/toxics9100248

**Published:** 2021-10-04

**Authors:** Walubita Mufalo, Pawit Tangviroon, Toshifumi Igarashi, Mayumi Ito, Tsutomu Sato, Meki Chirwa, Imasiku Nyambe, Hokuto Nakata, Shouta Nakayama, Mayumi Ishizuka

**Affiliations:** 1 Division of Sustainable Resources Engineering, Graduate School of Engineering, Hokkaido University, Sapporo 060-8628, Japan; 2 Division of Sustainable Resources Engineering, Faculty of Engineering, Hokkaido University, Sapporo 060-8628, Japan; tangviroon.p@gmail.com (P.T.); tosifumi@eng.hokudai.ac.jp (T.I.); itomayu@eng.hokudai.ac.jp (M.I.); tomsato@eng.hokudai.ac.jp (T.S.); 3 IWRM Centre/Geology Department, School of Mines, The University of Zambia, Lusaka 32379, Zambia; meki.chirwa@gmail.com (M.C.); inyambe@gmail.com (I.N.); 4 Faculty of Veterinary Medicine, Hokkaido University, Kita 18, Nishi 9, Kita-Ku, Sapporo 060-0818, Japan; hokuto.nakata@vetmed.hokudai.ac.jp (H.N.); shouta-nakayama@vetmed.hokudai.ac.jp (S.N.); ishizum@vetmed.hokudai.ac.jp (M.I.)

**Keywords:** contamination, lead, zinc, bio-accessibility, leaching, Kabwe, Zambia

## Abstract

Zambia’s Kabwe mine wastes (KMWs) are responsible for contaminating the surrounding soil and dust in the Kabwe district. Unfortunately, these wastes arise from the historical mining activities of lead (Pb) and Zinc (Zn), which lacked adequate waste management strategies. As a result, potentially toxic elements (PTEs) (Pb and Zn) spread across the Kabwe district. To assess the soil pollution derived from previous mining activities, we studied topsoil samples (*n* = 8) from the school playground soils (SPs). In this study, the degree of contamination, geochemical partitioning, and leachability, coupled with the release and retention of Pb and Zn, were studied. The SPs were classified as extremely enriched (EF > 40) and contaminated with Pb (I_geo_ > 5). On average, Pb (up to 89%) and Zn (up to 69%) were bound with exchangeable, weak acid-soluble, reducible and oxidizable phases, which are considered as ’geochemically mobile’ phases in the environment. The leachates from the soils (*n* = 5) exceeded the Zambian standard (ZS: 190:2010) for Pb in potable drinking water (Pb < 0.01 mg/L). Furthermore, the spatial distribution of Pb and Zn showed a significant reduction in contents of Pb and Zn with the distance from the mine area.

## 1. Introduction

Kabwe is a district in the central province of Zambia, which has geology dominated by carbonate-hosted Pb-Zn deposits [1]. Kabwe town is considered one of the most polluted cities globally due to the historical mining of lead (Pb) and Zinc (Zn) [2]. Mining activities between 1902 and 1994 produced approximately 0.8 Mt and 1.8 Mt of Pb and Zn, respectively [3]. The deposits are composed of massive sulfides ore bodies surrounded by secondary mineralization oxide zones of silicate ore (willemite; Zn_2_SiO_4_), cerussite (PbCO_3_), quartz (SiO_2_), smithsonite (ZnCO_3_), goethite (FeOOH), hematite (Fe_2_O_3_), and metal-bearing; vanadates, phosphates, carbonates of Zn, Pb, and V [1,3]. After nearly 90 years of mining without any environmental regulations, mining activities ceased in 1994 due to the depletion of the mineral resources. As a result, huge stockpiles of Kabwe Mine Wastes (KMWs) were disposed of and left unattended after decommissioning the mines in 1994. The profound KMWs consist of Zinc leach plant residues (ZLPRs) [4] and slag heaps [5].

Kabwe town has a semi-arid/barren topography. Thus, dust generation and transportation of potentially toxic elements (PTEs) from KMWs to the surrounding environment is high. Although many researchers have proposed immobilization techniques to reduce contamination in the town [6,7,8], pollution of the surrounding soils with PTEs is inevitable because of the dust dispersion from KMWs [9]. Generally, PTEs are bioaccessible through inhalation, intentional/accidental ingestion of contaminated soil, or dust [10,11]. Depending on age groups, pica behavior is higher in children than adults because of their hand-to-mouth etiquette [11,12]. The U.S Environmental Protection Agency (EPA) estimates that a typical child up to 84 months can swallow 100 mg of contaminated soil/day [13]. Moreover, a recent study in Kabwe revealed high Pb contents in the infant’s feces with an overall average value of 39.2 mg/kg, ranging from 0 to 256.7 mg/kg (dry weight) [14]. In addition, the children within the vicinity of the mine area of Kabwe have constantly recorded high blood lead levels (BLL) (BLL > 5 µg/dL) [15,16]. 

Since children are the most affected, one potential exposure source of Pb intoxication are school playgrounds soils (SPs). Most of the playfields for the children in Zambia are characterized by bare land and insufficient lawn maintenance. Thus, play activities are depicted by the substantial generation of dust from the soil. Since children are typically fond of playing in the SPs, there are several exposure pathways in which the PTEs can enter the body, i.e., ingestion or inhalation of soil settled on play equipment or dust adhered to hands, fingers, or clothes [17,18]. Prolonged exposure to this environment may lead to the uptake of PTEs into the respiratory and digestive systems. Consequently, elevated blood Pb is detrimental to human health and may cause severe nervous system problems, retardation, behavioral disorders [11], and formation of porous bones due to substitutions (Pb apatite’s) inside the bones.

Previous research works on Kabwe have reported high contents of Pb (>20,000 mg/kg) in the topsoils of the former smelter and processing plant for Pb and Zn [6]. However, detecting high contents of PTEs in the soil, in particular, Pb and Zn, does not necessarily mean the high risk of contaminants in the environment or bioavailability into the body. Instead, this depends on the leachability of the heavy metals under various environmental conditions [19]. Furthermore, the bioaccessible and bioavailable Pb and Zn in the soil depends on the binding and mineralogical forms in which they exist in [20,21]. Thus, understanding their specific characteristics in the binding states leads to their bio-accessibility in the soil because the release and retention mechanisms of heavy metals in the soils are influenced by the solubility-controlling mineral phases [22].

Therefore, the purpose of this study was to determine the extent of pollution, leachability, and the solid-phase partitioning of Pb and Zn in the SPs, because the playgrounds may be potential sources of PTEs for the children in Kabwe.

## 2. Materials and Methods

### 2.1. Site Description and Sampling Location

**Figure 1:**
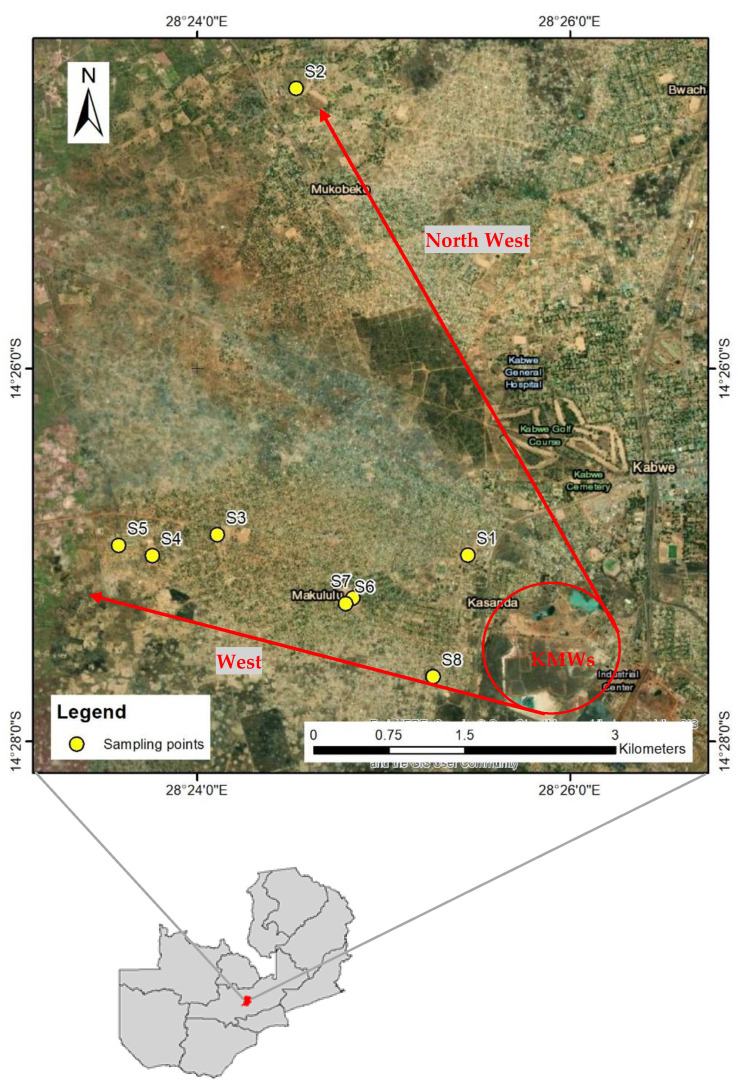
A map of Zambia with the sampling locations of Kabwe school playground soils (SPs): northwest direction (S1 and S2) and the west direction (S3, S4, S5, S6, S7, and S8) of Kabwe Mine Wastes (KMWs).

Figure 1 shows the location of Kabwe and the sampling points of the SPs. The soil samples were collected in the dry season from the west and northwest of the KMWs. The prevailing winds are usually downwind of the KMWs, to the west-northwest direction [6]. A total of 8 soil samples were collected from a community ground in Kasanda area (S1), Makululu primary school (S2), Malumbo community ground (S3), Makululu day secondary (S4), Makululu day secondary garden (S5), David Ramushu secondary school (S6), David Ramushu secondary school garden (S7), and Kebar Christian academy (S8). All the sampling points were approximately within a radius of 10 km from the mine area. Using a stainless-steel hand shovel, soil samples were collected from topsoil (about 5 cm depths) with 3 to 4 mixing points for each sample. These samples were stored in airtight vacuum bags and then shipped to Hokkaido University, Japan. The soil samples were then dried at room temperature before characterization, sequential extraction, and leaching experiments. 

### 2.2. Soil Characterization 

Mineralogical and chemical properties of the soils were conducted using pressed powders of the SPs (<50 µm) and analysed using X-ray diffractometer (XRD) (MultiFlex, Rigaku Corporation, Tokyo, Japan) and X-ray fluorescence spectrometer (XRF) (Spectro Xepos, Rigaku Corporation, Tokyo, Japan) respectively. The SPs’ particle size distribution was analyzed using the laser diffraction (Microtrac^®^ MT3300SX, Nikkiso Co. Ltd., Osaka, Japan). The total organic carbon content was calculated by the difference between total carbon content and inorganic carbon content measured by a total carbon analyzer with a solid sample combustion unit (TOC-VCSH-SSM-5000A, Shimadzu Corporation, Kyoto, Japan). 

### 2.3. Determination of Pollution Indices 

Two pollution indices were employed to calculate the extent of contamination in the SPs, i.e., geo-accumulation index (I_geo_) and enrichment factor (EF). The I_geo_ quantifies the degree of anthropogenic contamination. The index was introduced by Muller [23], given by the following equation.

I_geo_ = log_2_(C_n_/1.5B_n_) (1)

where, C_n_ is the content of an examined element in the SPs and B_n_ is the background value of the element in the studied area. This study selected the median soil background values in Sub-Sahara Africa [24] because it gives a more representative extent of pollution. The value of 1.5 is a constant introduced to normalize the variances in background levels. Seven categories based on the level of contamination are classified as: class 0,uncontaminated (I_geo_ < 0); Class 1, uncontaminated to moderately contaminated (0 < I_geo_<1); Class 2, moderately contaminated (1 < I_geo_ <2); Class 3, moderately to heavily contaminated (2 < I_geo_ <3); Class 4, heavily contaminated (3 < I_geo_ <4); Class 5, heavily to extremely contaminated (4 < I_geo_ <5); and Class 6, extremely contaminated (I_geo_ > 5).

The EF differentiates between metals originating from human activities and natural processes. The EF was calculated as the ratio of the content of the metal in the sample to the content of the same metal in the earth crust using the modified formula from Buat-Menard and Chesselet [25], as shown below:

EF = (M_x_∗Ti_b_)/(M_b_∗Ti_x_) (2)

where, M_x_ is the content of the measured sample, Ti_b_ is the background content of titanium (Ti) in the earth crust, here we chose Ti as a reference element for normalization because it is conservative; historic mining activities may not influence Ti in the SPs compared to Al or Fe. Meanwhile, M_b_ is the background content of the targeted element in the earth crust, while T_x_ is the measured content of the reference value in the sample. The earth crust content values were taken from Turekian and Wedepohl [26]. The calculated values were based on the classification of enrichment as: deficiency to minimal enrichment (EF < 2); moderate enrichment (2 < EF < 5); significant enrichment (5 < EF < 20); very high enrichment (20 < EF < 40); and extremely high enrichment (EF > 40) [27].

### 2.4. Geochemical Modeling and Multivariant Statistical Analysis

PHREEQC; Version 3, United states, Geological Survey, Sunrise Valley Drive Reston, USA, was used to calculate the saturation indices (*SIs*) for the possible mineral phases controlling the release and retention of Pb and Zn in the SPs [28]. The thermodynamic properties were taken from the MINTEQ.V4.DAT database. Principal component analysis (PCA) was used for finding the correlations between the metal content or metal ion concentration and several environmental leaching conditions. 

### 2.5. Batch Leaching Experiments

The batch leaching experiments were conducted by mixing 15 g of soil sample and 150 mL of deionized (DI) water in a 300-mL Erlenmeyer flask followed by lateral reciprocating shaking speed of 200 rpm for 6 h. After the predetermined shaking time, the pH, oxidation-reduction potential (ORP) and electrical conductivity (EC) were measured followed by centrifugation of the suspension using a centrifuge (Sigma 3K30 laboratory centrifuge, Osterode, Germany) for 30 min at a speed of 3500 rpm. The leachates from the experiments were collected by filtration through 0.45 Millex^®^ filters (Merck Millipore, Burlington, MA, USA). The leachates were preserved by 1% nitric acid (HNO_3_) before chemical analysis to maintain the target elements in dissolved forms. For accuracy and precision, all batches were performed in triplicates. The concentrations of major ions were then analyzed by inductively coupled plasma atomic emission spectroscopy (ICP-AES) (ICPE-9000, Shimadzu Corporation, Kyoto, Japan) (margin of error = ± 2~3%, determination limit 0.01–0.001 mg/L). All the chemicals used for conducting the experiments were reagent grade (Wako Pure Chemical Industries Ltd, Osaka, Japan).

### 2.6. Sequential Extraction

The sequential extraction experiment was based on the earlier works of Tessier et al. [29] and modifications of the procedure by Marumo et al. [30]. The procedure partitions Pb and Zn into five phases: (1) exchangeable, (2) carbonates, (3) iron and manganese oxides, (4) organic matter and sulfide minerals, and (5) residual. The extraction procedure is summarized in Table 1.

The extraction was performed on 1g of the SPs sample (<2mm) mixed with a predetermined volume of the extractant for each step. The leachate was separated from the residue by centrifugation of the suspension at 3000 rpm for 30 min. Between each step, the residue was washed with 20 mL of DI water, which was combined with the leachate of the extraction step and diluted to 50 mL. The diluted solution was filtrated through 0.45 Millex^®^ filters, and kept in polypropylene bottles before chemical analysis. The samples were analyzed using inductively coupled plasma atomic emission mass spectrometry (ICP-MS) (ICAP Qc, Thermo Fisher Scientific, Waltham, MA, USA).

## 3. Results 

### 3.1. Chemical Composition and Mineralogy of SPs

Table 2 illustrates the major chemical composition, total organic carbon (TOC) and trace elements (Pb and Zn) of the SPs. The samples were mainly dominated with SiO_2_ (56.1–83.7 wt%), Al_2_O_3_ (6.4–29.7 wt%), and Fe_2_O_3_ (2.2–7.6 wt%). The TOC for the samples ranged from 0.3 to 5 wt%. The SPs were classified according to the United States Department of Agriculture (USDA) soil classification, as either sandy loam (S1, S5, S6, and S7), silt loam (S3, S4, and S8), or loam (S2) (Table S1). High contents of trace elements of Pb (265–3320 mg/kg) and Zn (359–2600 mg/kg) were recorded. Although XRD did not detect Pb-and Zn-bearing minerals, the possible mineral phases might be attributed to anglesite (PbSO_4_), and zinkosite (ZnSO_4_) [7]. The contents of Pb and Zn are much higher than the average background contents in Sub-Sahara Africa; Pb (37 mg/kg) and Zn (29 mg/kg) [24].

Figure S1 shows XRD patterns of the SPs. Quartz (SiO_2_) is the primary mineral in all the SPs. Noticeable kaolinite peaks (Al_2_SiO_5_(OH)) were detected in samples S1, S2, S3, S4, and S5. This is consistent with the high content of Al_2_O_3_ (Table 1) and the clay fractions in the samples (S1, S2, S3, S4, and S5) (Table S1).

Table 3 shows PCA multivariant correlations in the SPs using variables in Table 1. PCA 1, PCA 2, and PCA 3 accounted for 82% of the total chemical composition of the SPs. PCA 1 showed that the Pb and Zn were associated with TOC and S with a 42% variance. This means that Pb and Zn had a close relationship with these chemical components in the SPs. PCA 2 and PCA 3 accounted for 21 and 19%, respectively. The high loadings of Zn and Al_2_O_3_ from PCA 2 indicate Zn’s associations with the phyllosilicate minerals present in deposited material from old smelting activities and dust dispersion from KMWs. PCA 3 is mainly attributed to the geogenic chemical components (CaO, Fe_2_O_3,_ and MnO) controlling the characteristics of the SPs such as pH, and release, and retention of Pb and Zn.

Table S2 shows the Pearson correlation matrix for the chemical composition of the SPs. The results indicated a significant correlation between TOC and S (*r* = 0.8, *p* < 0.05). Pb showed high correlations with TOC (*r* = 0.8, *p* < 0.05) and S (*r* = 0.7, *p* < 0.05). On the other hand, Zn showed lower correlation with TOC (*r* = 0.6, *p* > 0.05) and S (*r* = 0.4, *p* < 0.05). This maybe because zinc sulfide (ZnS) oxidizes faster on air than galena (PbS); a problem known from the sediments of the Wadden sea in Germany. 

### 3.2. The Extent of Pollution and Particle Size Distribution of the SPs

Table S3 summarizes I_geo_ and EF. The SPs from S1, S3, S7 and S8 showed ‘extremely high enrichment’ of Pb (EF > 40). Meanwhile, S2, S5 and S6 had ‘very high enrichment’ of Pb (20 < EF < 40). The SPs (S4) had the lowest enrichment of Pb (5 < EF < 20, indicating a significant enrichment). However, only (S1) showed ‘very high enrichment’ for Zn (20 < EF < 40), while S2, S3, S6 and S7 indicated ‘significant enrichment’ of Zn (5 < EF < 20). The order of enrichment followed S1 > S8 > S3 > S7 > S6 > S2 > S5 > S4 for Pb and S1 > S8 > S7 > S6 > S2 > S3 > S5 > S4 for Zn. 

The I_geo_ for SPs indicated that S1, S2, S3, S6, S7, and S8 were ‘extremely contaminated’ with Pb (I_geo_ > 5). The S4 and S5 were (heavily:3 < I_geo_ < 4) and (heavily to extremely: 4 < I_geo_ < 5) contaminated with Pb respectively. Meanwhile, S1, S2, S6, S7 and S8 were ‘extremely contaminated’ with Zn. The order of contamination followed S1, S8 > S7 > S3, S2 > S6 > S5 > S4 for Pb and S1 > S8, S2 > S7 > S6 > S3 > S5 > S4 for Zn. The pollution indices shows that the SPs were highly enriched and contaminated with PTEs.

**Figure 2:**
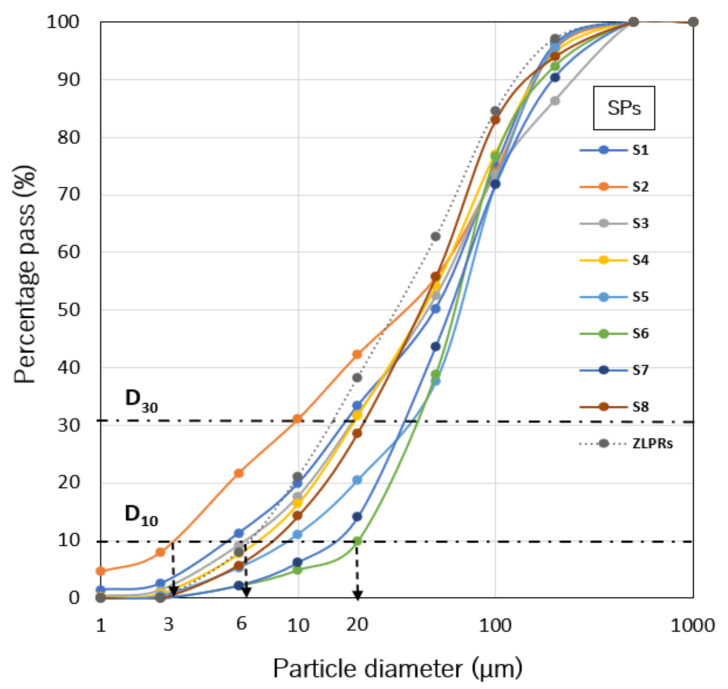
Particle size distribution of SPs and the zinc leach plant residues (ZLPRs) with their 10% and 30% diameters D_10_ and D_30_.

Figure 2 shows the particle size distribution of the SPs and predominate KMWs, i.e., ZLPRs. The residues had fine particles (<6 µm is a 10% effective diameter (D_10_)). Kabwe is characterized by bare land and a windy, dry season [6]. Therefore, particles (<20 µm) are likely to become aerosol from the KMWs; consequently, finer particles are transported further [31]. Moreover, S2, the furthest site from the KMWs had ‘very high enrichment’ of Pb. The simulation results at S2 by Nakamura et al. [9] suggest that prevailing winds in the northwest, north-northwest, and north direction from the KMWs might have influenced the deposition of smaller particles to the area. 

### 3.3. Leaching Characteristics of SPs

**Figure 3:**
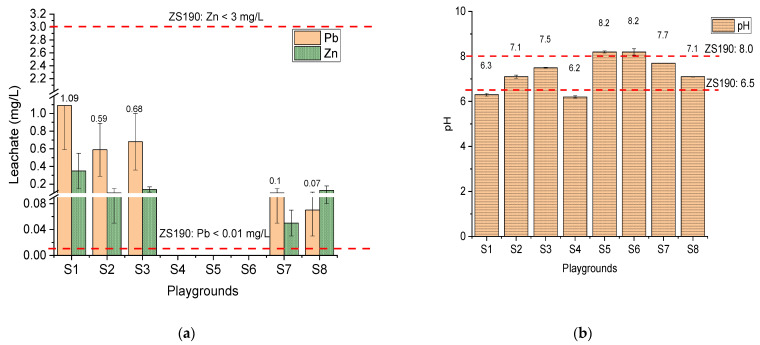
Leachate characteristics for the SPs: (**a**) water-soluble Pb and Zn (**b**) pH. The dotted line represents permissible limits for drinking water standards in Zambia (ZS190.2010).

Figure 3 shows the leachate characteristics of the SPs based on the standard leaching tests. The leachates were compared with the Zambia Bureau of standards for drinking water quality (ZS.190.2010). The quality prescribes requirements for drinking water suitable for human consumption. The SPs from S4, S5, and S6 had no leachable Pb, while S1, S2, S3, S7, and S8 had leaching concentrations of Pb above the permissible limit for drinking water in Zambia (Pb < 0.01 mg/L) (Figure 3a). Meanwhile, Zn was within the tolerable limit for all the SPs (Zn < 3 mg/L). The pH of all the SPs ranged from slightly acidic to alkaline conditions (pH 6.2–8.2). The pH values of the leachates from S1, S4, S5, and S6 exceeded the Zambian drinking water standard (pH 6.5–8.0), while those from S2, S3, S7, and S8 were within the permissible limits (Figure 3b). 

Table 4 shows the results from PCA analysis using the leachate concentration. PCA 1 and PCA 2 accounted for 79% of the total variance, with high loadings of Al, Si, Fe, Pb, SO_4_^2^^−^, pH, EC, Eh, Mg and Zn. The PCA loadings of alkalinity, positive pH, and Ca in PCA 3 (17% variance) indicates the chemical components controlling the buffering effects of the SPs. This is consistent with geogenic CaO in PCA 3 from the chemical composition of the SPs. The high loadings of SO_4_^2−^, Mg, EC, and Eh in PCA 2 accounted for 27% of the co-existing ions controlling the EC of the leachate in SPs. 

### 3.4. Partitioning of Pb and Zn in the SPs

**Figure 4:**
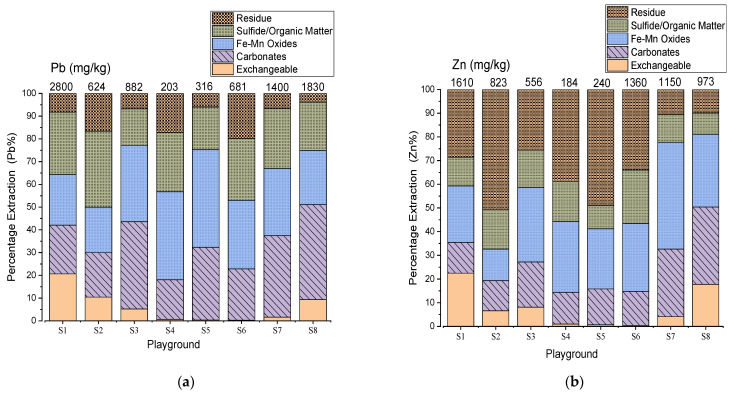
Solid-phase partitioning of Pb (**a**) and Zn (**b**) in SPs. The values on top of each sample represent the total content (mg/kg) from the extraction of the five phases. The phases are expressed in percentage of the total content.

The sequential extraction patterns of Pb and Zn are shown in Figure 4. The solid-phase partitioning of Pb in the SPs ranged from 0.3 to 21% for the exchangeable fraction, weak acid soluble (17 to 42%), reducible (20 to 43%), oxidizable (16 to 33%), and residue (4 to 20%) (Figure 4a). Meanwhile for Zn, exchangeable (0.4 to 23%), weak acid soluble (13 to 33%), reducible (13 to 45%), oxidizable (9 to 23%) and residue (10 to 51%) (Figure 4b).The Pb in the SPs was mainly bound with carbonates, reducible and oxidizable phases while Zn showed strong association with reducible and residue fractions. 

The SPs of S4, S5, and S6 with the undetected water-soluble Pb corresponded to the exchangeable phase; 0.6% (S4), 0.4% (S5) and 0.3% (S6) (Table S4). On the other hand, Zn was mainly in the reducible phase of 30% (S4), 25% (S5), and 29% (S6), and the residue phase of 39% (S4), 49% (S5), and 34% (S6) (Table S5). The highest partitioning for Pb in the SPs (S4, S5, and S6) was with the Fe and Mn oxides. 

Table S6 shows the labile phases (Phase 1; exchangeable + Phase 2; carbonates + Phase 3; Fe/Mn oxides) for Pb and Zn. The labile phases for Pb added up to 50 to 77%. Those for Zn added up to 33 to 81%. The results indicate that the mobile fractions were notably high for all the SPs. 

Figures S2 and S3 illustrate the leachable water-soluble (Pb and Zn) relationship with the exchangeable, labile phases and the total content. The water-soluble Pb showed a strong correlation with exchangeable phase (*r* = 0.8), labile phase (*r* = 0.6), and a lower correlation with the total content (*r* = 0.5). Meanwhile, the correlation for the water-soluble Zn showed (*r* = 0.9) with the exchangeable phase, *(r* = 0.5) with the labile phase and (*r* = 0.6) with the total content. The correlations certainly indicate the close relationship between the water-soluble Pb and Zn with the exchangeable phase.

### 3.5. The Distance of SPs from the KMWs

**Figure 5:**
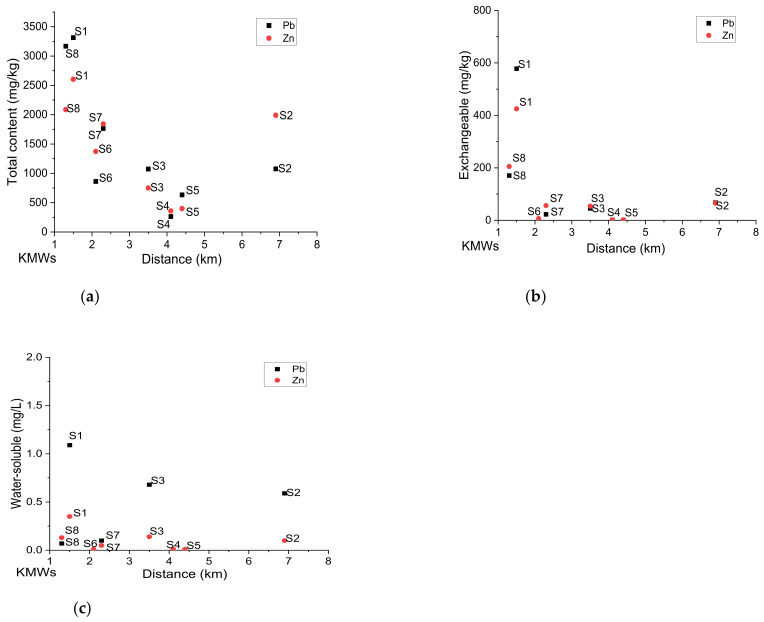
The distribution of Pb and Zn with the distance of each playground from Kabwe Mine Wastes (KMWs): (**a**) contents (XRF), (**b**) exchangeable and (**c**) water-soluble.

Figure 5 shows the trend for the total content, exchangeable phase, and the water-soluble Pb and Zn for each SPs site with the distance from the KMWs. The S1 and S8 are the nearest sites to the KMWs. Consequently, they had the highest contents of Pb and Zn, with S1 having 3320 mg/kg for Pb and 2600 mg/kg for Zn, while S8 records 3170 mg/kg for Pb and 2090 mg/kg for Zn (Figure 5a). The trend was similar to water-soluble and exchangeable phases (Figure 5b,c). There was a noticeable decrease as moving away from the KMWs. 

## 4. Discussion

The SPs have exceptionally high contents of Pb and Zn. Soil contamination in the SPs might be attributed to the deposition of aerosol particles to surrounding soils from the KMWs. Nakamura et al. [9] confirmed that the contents of Pb generally increased with the amount deposited; also, the Pb content in the SPs and the simulated amount of deposition decreased with distance from the source. This is consistent with the high enrichment and contamination of Pb in the soils near the mine area (S1 and S8). The results of the substantial enrichment in S1 and S8 were consistent with the profound intensity of contamination in the SPs near the KMWs. 

However, the discrepancies in the contents and leaching of Pb and Zn in the SPs might be attributed to the volume of aerosols deposited, particle size distribution and environmental conditions controlling the mobilities of the PTEs. Moreover, the mobility of PTEs such as Pb and Zn in groundwater, mine tailings, and contaminated soils are extensively influenced by the pH, precipitation, redox conditions, and adsorption/desorption reactions [32]. Regardless, the enrichment of Pb was exceptionally higher than Zn in all the SPs. In comparison with other playgrounds for different cities in the world, the contents of Pb and Zn from Kabwe SPs were higher than those of Warsaw and Bydgoszcz, Poland (Pb up to 167 mg/kg, Zn up to 57.4 mg/kg [33], Beijing, China (Pb up to 207.5 mg/kg, Zn, up to 196.9 mg/kg [34], Ibadan, Nigeria (Pb up to 58 mg/kg, Zn up to 460 mg/kg [35] and Hong Kong, country parks (Pb up to 124 mg/kg, Zn up to 136 mg/kg [36].

Figure 3a showed that the Pb in the leachates from S1, S2, S3, S7, and S8 were above the permissible limit for drinking water in Zambia. Water consumption from such an environment may be detrimental to human health because some communities in Kabwe rely on groundwater from boreholes. The release of Pb into the environment may be attributed to sulfide oxidation [21]. This is explained by the SO_4_^2−^ in PCA 2 for the leachate concentration. The high loadings of SO_4_^2−^, EC, and Eh suggest the sulfide minerals oxidation in the SPs. Moreover, Silwamba et al. [4] pointed out that in the leaching system for ZLPR, the SO_4_^2−^ was attributed to the soluble minerals phases, anglesite (PbSO_4_), zinkosite (ZnSO_4_)_,_ and gypsum CaSO_4_^.^2H_2_O. 

Table S7 shows the PHREEQC simulation for the possible mineral phases that could affect the release and retention of PTEs in the SPs. The results shows that the *SIs* for CaSO_4_^.^2H_2_O (−3.45 to −1.55) and PbSO_4_ (−2.41 to −1.43) implied the dissolution of these mineral phases. Consequently, suggesting that the release Pb in the SPs may be from the dissolution of PbSO_4_. However, the *SIs* of goethite (Fe(OH)_3_; 4.6 to 7.94) and those of ferrihydrite (FeOOH; 1.85 to 5.22) showed that precipitation was thermodynamically favored after leaching. This explains why no leachable Pb, and Zn were detected in S4, S5 and S6, meaning that the available PTEs in the SPs might have been redistributed and adsorbed with hydrous ferric oxides (HFOs). Moreover, HFOs have an excellent affinity for PTEs such as Pb, Cu, Cd, Zn, Ni, and metal oxyanions such as Cr, Sb, and As [37,38]. This is consistent with batch leaching results; no Fe was detected in the S4, S5, and S6 (Table S8). 

Fractionation of Pb by sequential extraction confirmed the strong associations between Pb with the Fe/Mn oxides and sulfide/organic phases. This means that Pb was relatively stable after deposition [39]. Therefore, the Pb in the Fe/Mn phase might have been attributed to weathering of PbS. The Pb bound to the oxidizable (sulfide/organic) phases was higher than Zn, which is consistent with the high correlation of Pb and S for the chemical composition of the soils. Although the SPs were characterized by bare land and little vegetative cover, the strong correlations of Pb with TOC suggest that some of the Pb might be retained with the organic phases [40]. Agnieszka et al. [41] also highlighted the strong correlations of Pb, Cu, Ni, and Cr with TOC in the organic phase. 

The mobile fractions for the labile phases (exchangeable + carbonates + Fe/Mn oxides) for Pb and Zn were notably high for all the SPs. Generally, it is typically assumed that the greater the labile percentage, the greater the potential for bioavailability in the body [42]. Therefore, the high bioaccessible fractions of PTEs in the labile phases significantly increase the bioaccessibility of the ingested PTEs, thereby posing a serious threat to the respiratory and digestive systems for the children because a substantial amount of PTEs might be absorbed into the bloodstream. 

The correlations between the leachable water-soluble Pb and Zn with the exchangeable, labile phases and the total content suggests that the release of Pb and Zn into the environment from the SPs was mainly attributed to the exchangeable phase and not so much from the total content of Pb and Zn (Figures S2 and S3). In addition, the Pb bound to carbonate phase is usually influenced by the pH of the environment; for example, under gastric conditions, cerussite (PbCO_3_) might significantly dissolve in the acidic conditions [43], which increases the bioaccessibility of ingested PTEs. However, the present study could not identify the host minerals for the PTEs; electron microprobe analysis might be a possible approach to identifying the mineral phases [44].

## 5. Conclusions

The leachability, solid-phase partitioning and contamination assessment of the SPs were conducted in Kabwe SPs near the mine area. The anthropogenic contamination and enrichment of Pb and Zn in the SPs can be traced back to the old mining activities. The SPs were extremely enriched with PTEs. The Pb and Zn in the SPs were mainly bound with the carbonates, reducible and oxidizable phases. The bioaccessible fractions of the PTEs for the labile phases were exceptionally high for all the SPs. 

Thus, the SPs are potential sources of PTEs for the children in Kabwe. In general, the human health risk associated with inhalation/ingestion of PTEs from the SPs is high. Therefore, revegetation, soil turning, and watering the SPs to suppress dust generation could be some short-term measures to reduce the high bioaccessibility of PTEs in the SPs. 

## Figures and Tables

**Table 1:** Procedure for the Determination of the Solid-Phase Partitioning of Pb and Zn.

Phase	Extractant	L/S Ratio (mL/g)	Temperature (°C)	Duration (h)	Speed (rpm)	Extracted Phase
1	1 M MgCl_2_ at pH 7	20/1	25	1	200	Exchangeable
2	1 M CH_3_COONa at pH 5	20/1	25	5	200	Carbonates
3	0.04 M NH_2_OH·HCl in 25% acetic acid	20/1	80	5	120	Reducible
4	0.04 M NH_2_OH·HCl in 25% acetic acid; 30% H_2_O_2_; 0.02 M HNO_3_	36/1	80	5	120	Oxidizable
5	60% HNO_3_	20/1	120	1		Residue

**Table 2:** Chemical Composition of the Major Components and the Trace Elements in the School Playground Soils (SPs).

				SPs				
	S1	S2	S3	S4	S5	S6	S7	S8
SiO_2_ (wt%)	56.4	56.1	72.2	67.7	60.7	69.9	75.4	83.7
TiO_2_ (wt%)	0.9	1.6	0.8	1.4	1.0	1.0	1.3	1.0
Al_2_O_3_ (wt%)	22.8	29.7	16.6	23.9	15.9	8.5	15.2	6.4
Fe_2_O_3_ (wt%)	7.2	4	7.6	6.2	6.9	6.2	4	2.2
MnO (wt%)	0.54	0.10	0.10	0.06	0.24	0.17	0.11	0.09
MgO (wt%)	0.8	1.9	1.2	2.8	2.3	0.9	1	0.6
CaO (wt%)	0.3	0.6	1.2	0.1	2.2	2	0.7	0.8
Na_2_O (wt%)	<0.1	<0.1	<0.1	<0.1	<0.1	<0.1	<0.1	<0.1
K_2_O (wt%)	0.9	0.8	1.1	1.1	0.7	0.7	1.5	0.7
P_2_O_5_ (wt%)	0.26	0.14	0.17	0.08	0.06	0.12	0.26	0.24
S (wt%)	0.2	0.07	0.07	0.05	0.06	0.2	0.15	0.93
TOC (wt%)	2.46	1.86	2.79	0.52	0.33	0.77	1.55	5.02
Pb (mg/kg)	3320	1080	1070	265	633	863	1770	3170
Zn (mg/kg)	2600	1990	750	359	399	1370	1840	090

**Table 3:** Principal Component Analysis (PCA) for the Chemical Composition of the SPs, Including Total Variance, Eigenvalue, and Cumulative Frequency.

	PCA1	PCA2	PCA3
SiO_2_ (wt%)	0.24	−0.35	−0.31
Al_2_O_3_ (wt%)	−0.22	**0.49**	−0.04
Fe_2_O_3_ (wt%)	−0.24	−0.01	**0.44**
MnO (wt%)	0.07	0.25	**0.55**
MgO (wt%)	−0.37	0.04	−0.17
CaO (wt%)	−0.09	−0.47	0.25
K_2_O (wt%)	−0.02	0.21	−0.25
P_2_O_5_ (wt%)	0.37	0.23	0.02
S (wt%)	**0.36**	−0.18	−0.12
TOC (wt%)	**0.37**	−0.01	−0.07
Pb (mg/kg)	**0.39**	0.19	0.16
Zn (mg/kg)	**0.32**	**0.33**	0.08
Eigenvalue	5.47	2.68	2.45
Variance (%)	42.0	20.6	18.8
Cumulative	42.0	62.6	81.5

Bold values indicate high loading of the chemical components.

**Table 4:** Principal Component Analysis (PCA) for Water-Soluble Pb and Zn, including Total Variance, Eigenvalue, and Cumulative Frequency.

	PCA 1	PCA 2	PCA 3
pH	−0.24	**−0.31**	**0.38**
Eh (mV)	0.2	**0.42**	−0.29
EC (mS/cm)	−0.27	**0.36**	0.24
alkalinity	−0.24	−0.29	**0.38**
Ca (mg/L)	−0.34	0.12	**0.32**
Si (mg/L)	0.36	−0.02	0.29
Fe (mg/L)	**0.34**	−0.03	**0.32**
Al (mg/L)	**0.35**	−0.04	**0.31**
SO_4_^2-^ (mg/L)	−0.2	**0.46**	0.16
Pb (mg/L)	**0.36**	0.09	0.25
Zn (mg/L)	**0.28**	0.29	0.25
Mg (mg/L)	−0.19	**0.44**	0.19
Eigenvalue	6.19	3.26	2.01
Variance (%)	52	27	17
Cumulative	52	79	96

Bold values indicate high loading of the chemical components.

## Data Availability

Not applicable.

## Supplementary Materials

The following are available online at https://www.mdpi.com/article/10.3390/toxics9100248/s1, Table S1: A brief description of the type of soil samples collected, Figure S1: XRD patterns showing the major minerals found in the SPs, Table S2: Pearson correlation matrix for chemical composition for the soil samples. Marked correlation is significant at *p* < 0.05, Table S3: Pollution indices with the total average value across the SPs. Table S4 and Table S5: Solid-phase partitioning of Pb and Zn: mean content for each phase (mg/kg), standard deviation (SD), and percentage (%) partitioning for each phase in the SPs, Table S6: Labile % (Phase 1;exchangeable + Phase 2;carbonates + Phase 3;Fe/Mn oxides) for the SPs, Figure S2 and S3: Relationship between water-soluble Pb and Zn with (a) exchangeable phase, (b) labile Phases 1 + 2 + 3 and (c) total content, Table S7: Saturation indices (*SI*) of selected mineral phases controlling the release and retention of Pb and Zn in the selected SPs. Table S8: Leachate characteristics of the SPs, including other dissolved minerals.
