# Supplementary material for: Solid-Phase Partitioning and Leaching Behavior of Pb and Zn from Playground Soils in Kabwe, Zambia
Source: Toxics. 2021 Oct 4;9(10):248. doi: 10.3390/toxics9100248 (PMC8539481; doi:10.3390/toxics9100248)
Supplement: Supplementary file 1 [file toxics-09-00248-s001.zip › toxics-1380629-supplementary.pdf]

toxics

Supplementary Materials: Solid-phase partitioning and leaching behavior of Pb and Zn from playground soils in Kabwe, Zambia.

Walubita Mufalo, Pawit Tangviroon, Toshifumi Igarashi, Mayumi Ito, Tsutomu Sato, Meki Chirwa, Imasiku Nyambe, Hokuto Nakata, Shouta Nakayama and Mayumi Ishizuka

Summary

This supplement is divided into 4 sections, including 7 tables and 3 figures. It provides additional data on the following: (1) soil classification, X-ray powder diffraction (XRD), and Pearson correlation matrix for the soil samples (2) Degree of contamination (3) Solid-phase partitioning, labile mobile fraction % and water-soluble relations with the exchangeable phase, labile phase, and total contents of Pb and Zn in the SPs (4) PHREEQC analysis and batch leaching results

1. Soil classification, X-ray powder diffraction (XRD), and Pearson correlation matrix for the soil samples

Table S1. A brief description of the type of soil samples collected: Particle size distribution into clay, silt, and sand according to the United States Department of Agriculture for the size groups of mineral particles (USDA, 1987) [1].

Sample	Location	Type of Soil	Clay % (< 2 μm)	Silt % (2–50 μm)	Sand % (> 50 μm)
S1	Community ground in Kasanda	Sandy loam	2.51	47.9	49.6
S2	Makululu primary school	Loam	8	47.8	44.2
S3	Malumbo community ground	Silt loam	1.38	51.2	47.5
S4	Makululu day secondary	Silt loam	0.86	53.4	45.7
S5	Makululu day secondary garden	Sandy loam	0.42	37.2	62.3
S6	David Ramushu secondary school	Sandy loam	0	39.0	61.0
S7	David Ramushu secondary school garden	Sandy loam	0	43.8	56.2
S8	Kebar Christian academy	Silt loam	0.04	55.9	44.1

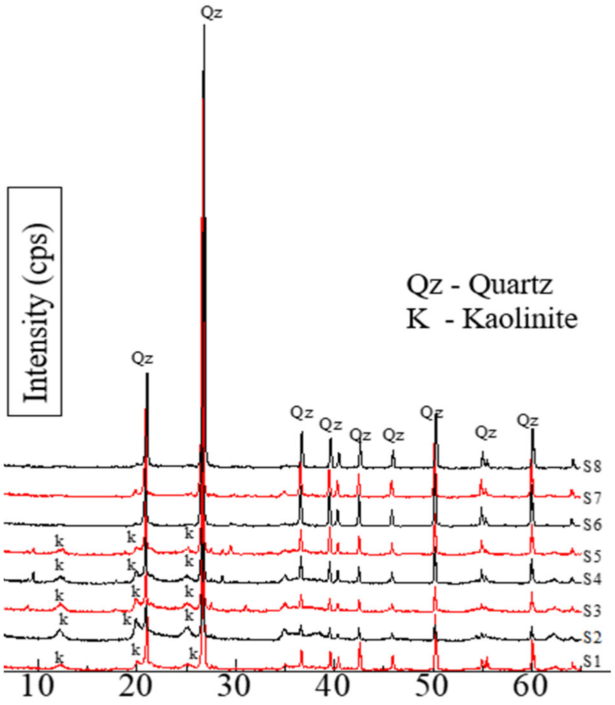

Figure S1. XRD patterns showing the major minerals found in the SPs.

Table S2. Pearson correlation matrix for chemical composition for the SPs. Marked correlation is significant at $p < 0.05$.

	SiO_2 (wt%)	TiO_2 (wt%)	Al_2O_3 (wt%)	Fe_2O_3 (wt%)	MnO (wt%)	MgO (wt%)	CaO (wt%)	K_2O (wt%)	P_2O_5 (wt%)	S (wt%)	TOC (wt%)	Pb (mg/kg)	Zn (mg/kg)
SiO_2 (wt%)	1.0												
TiO_2 (wt%)	-0.2	1.0											
Al_2O_3 (wt%)	-0.8	0.6	1.0										
Fe_2O_3 (wt%)	-0.5	-0.5	0.2	1.0									
MnO (wt%)	-0.6	-0.4	0.2	0.5	1.0								
MgO (wt%)	-0.4	0.5	0.6	0.3	-0.3	1.0							
CaO (wt%)	0.0	-0.4	-0.5	0.3	0.0	-0.1	1.0						
K_2O (wt%)	0.2	0.2	0.2	0.0	-0.2	0.0	-0.4	1.0					
P_2O_5 (wt%)	0.3	-0.2	-0.2	-0.4	0.3	-0.8	-0.4	0.4	1.0				
S (wt%)	0.7	-0.3	-0.6	-0.7	-0.1	-0.6	-0.1	-0.3	0.5	1.0			
TOC (wt%)	0.5	-0.3	-0.3	-0.5	0.0	-0.7	-0.3	-0.1	0.7	**0.8**	1.0		
Pb (mg/kg)	0.2	-0.4	-0.2	-0.4	0.5	-0.8	-0.4	-0.1	0.9	**0.7**	**0.8**	1.0	
Zn (mg/kg)	0.0	0.0	0.0	-0.5	0.4	-0.7	-0.4	0.0	0.8	**0.4**	**0.6**	**0.8**	1

2. Degree of Contamination

Table S3. Pollution indices with the total average value across the SPs.

Playgrounds	Enrichment factor (*EF*)		Geo-accumulation Index (I_{geo})	
	Pb	Zn	Pb	Zn
S1	141.4	23.4	6.9	6.4
S2	25.4	9.9	5.3	6.1
S3	53.2	7.8	5.3	4.6
S4	7.5	2.1	3.3	3.6
S5	23.2	3.1	4.6	3.7
S6	32.1	10.7	5.0	5.5
S7	50.3	11.1	6.0	5.9
S8	124.9	17.3	6.9	6.1
Average	**57.3**	**10.7**	**5.4**	**5.3**

3. Solid-phase partitioning, labile mobile fraction% and water-soluble relations with the exchangeable phase, labile phases, and total content of Pb and Zn in the SPs

Table S4. Solid-phase partitioning of Pb: mean content for each phase (mg/kg), standard deviation (SD), and percentage (%) partitioning for each phase in the SPs.

Pb	Phase 1			Phase 2			Phase 3			Phase 4			Phase 5		
SPs (*n* = 3)	Mean	SD	%	Mean	SD	%	Mean	SD	%	Mean	SD	%	Mean	SD	%
S1	578.2	59.1	20.7	599	84.6	21.4	622	60.8	22.3	767	81.2	27.5	229	27.6	8.2
S2	67.7	9.7	10.4	124	37.5	19.5	125	17.4	20.0	207	11.2	33.4	104	15.2	16.6
S3	45.5	5.7	5.2	341	78.3	38.4	295	35.5	33.6	140	2.6	16.1	59	3.8	6.8
S4	1.3	0.2	0.6	36	7.3	17.4	79	3.7	38.7	53	2.1	26.0	35	10.4	17.2
S5	1.2	0.1	0.4	101	18.2	31.9	135	10.8	43.0	59	0.2	18.8	19	2.2	6.0
S6	2.0	0.1	0.3	155	41.7	22.6	205	18.5	30.2	185	3.5	27.2	134	4.9	19.8
S7	22.9	4.4	1.6	533	84.2	35.8	412	38.3	29.5	370	54.3	26.4	93	0.5	6.7
S8	170.6	15.4	9.4	763	76.1	41.8	435	30.8	23.9	388	53.2	21.2	70	7.7	3.8

Table S5. Solid-phase partitioning of Zn: mean content for each phase (mg/kg), standard deviation (SD), and percentage (%) partitioning for each phase in the SPs.

Zn	Phase1			Phase 2			Phase3			Phase4			Phase 5		
SPs (*n* = 3)	Mean	SD	%	Mean	SD	%	Mean	SD	%	Mean	SD	%	Mean	SD	%
S1	362	55.1	22.5	198	15.1	13.0	383	6.1	23.8	194	19.0	12.0	460	8.4	28.6
S2	54	9.9	6.6	81	45.5	12.8	110	17.0	13.3	137	13.3	16.6	415	23.9	50.7
S3	45	7.5	8.1	98	14.9	19.1	176	34.5	31.5	88	9.3	15.8	141	22.8	25.6
S4	2	0.3	1.0	24	0.4	13.4	55	7.6	29.9	31	17.0	16.9	72	16.8	38.8
S5	2	0.8	0.7	37	1.9	15.1	57	5.1	25.4	25	19.9	9.8	118	27.9	49.0
S6	5	0.9	0.4	175	35.8	14.4	387	37.7	28.6	307	56.0	22.6	460	39.0	34.0
S7	47	9.6	4.1	293	62.3	28.6	517	71.9	45.1	138	43.9	11.9	117	14.7	10.4
S8	173	29.9	17.7	322	13.5	32.6	301	56.5	30.7	87	26.3	8.9	95	3.4	9.9

Table S6. Labile % (phase 1; exchangeable + phase 2; carbonates + phase 3;Fe/Mn oxides) for the SPs.

SPs	S1	S2	S3	S4	S5	S6	S7	S8
Pb	64	50	77	57	75	53	67	75
Zn	59	33	59	44	41	43	78	81

(a)

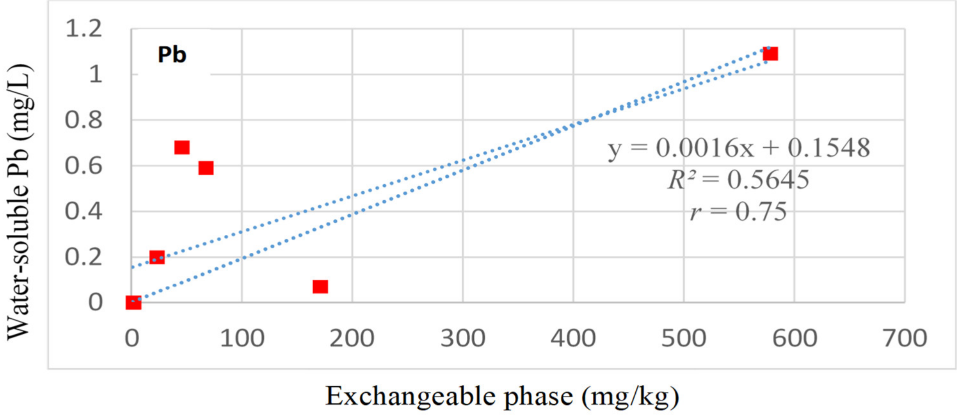

(b)

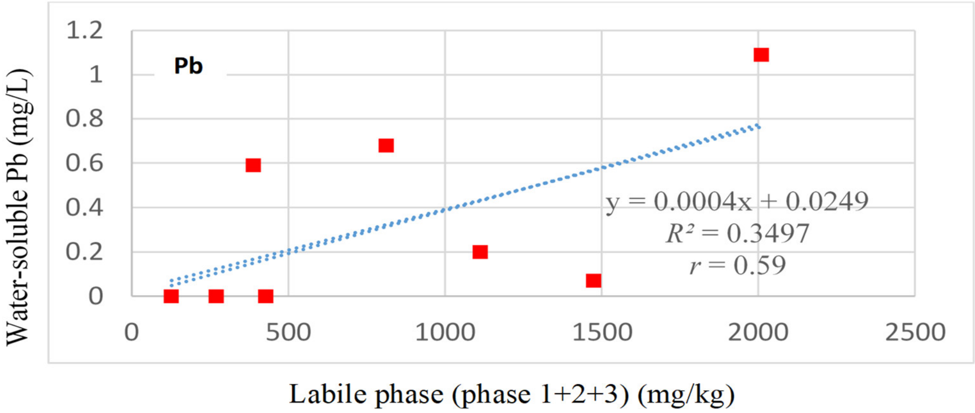

(c)

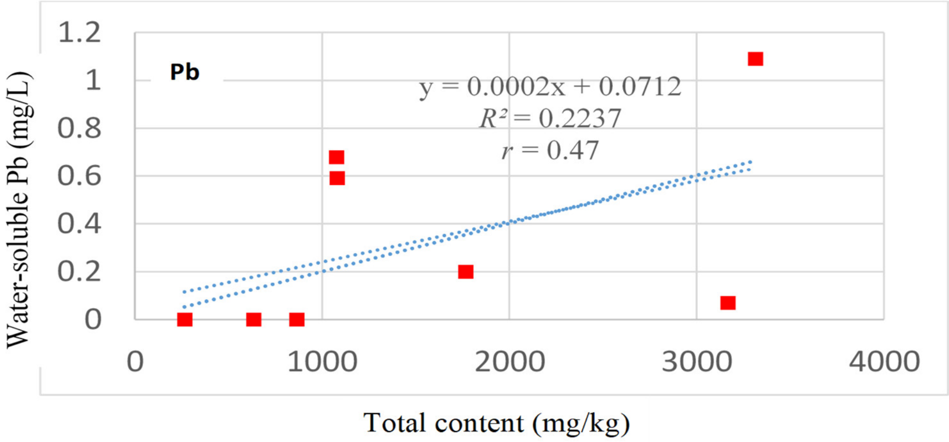

Figure S2. Relationship between water-soluble Pb with (**a**) exchangeable phase (**b**) labile phase 1+2+3 and (**c**) total content.

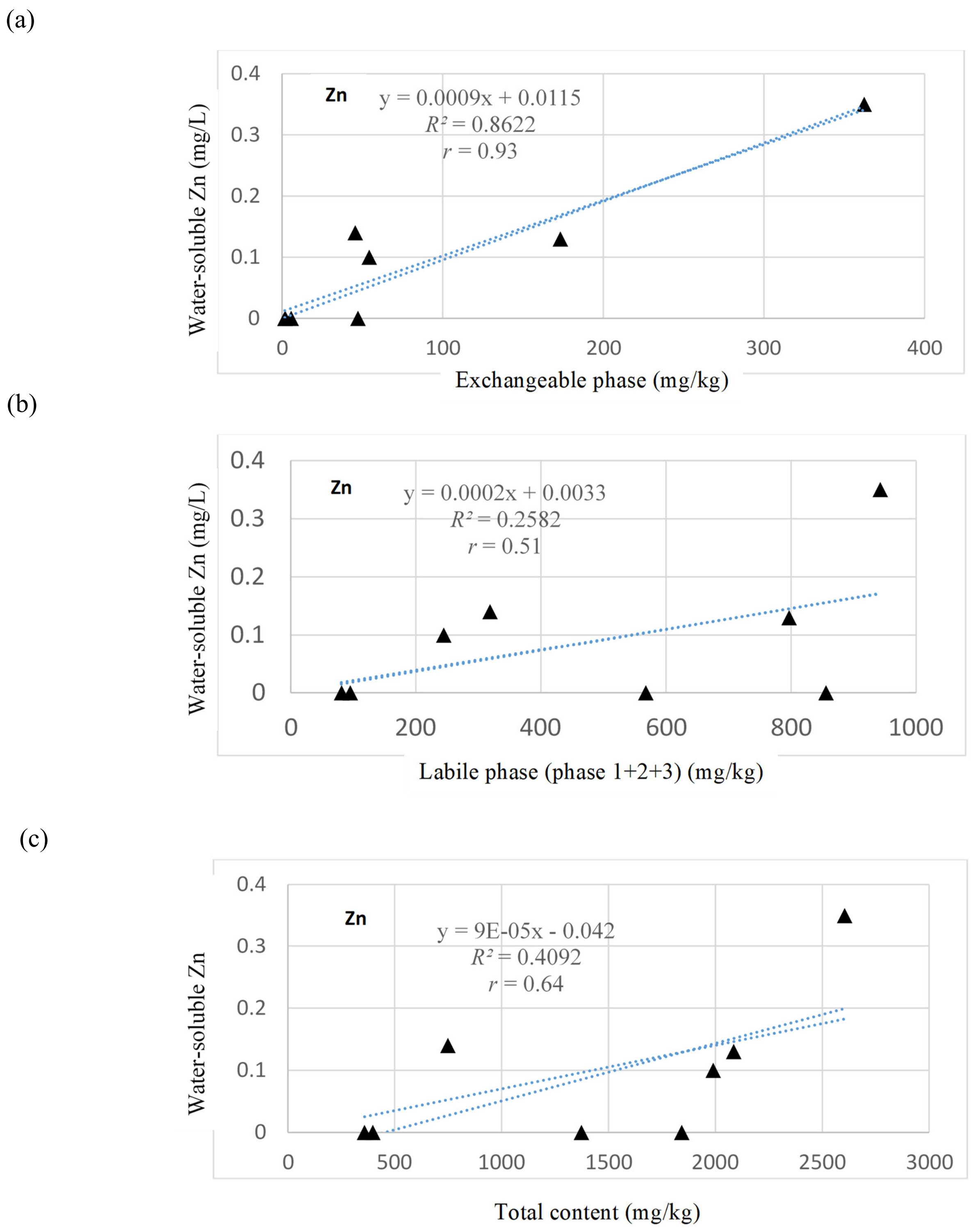

Figure S3. Relationship between water-soluble Zn with (**a**) exchangeable phase (**b**) labile phase 1+2+3 and (**c**) total content.

4. PHREEQC analysis and batch leaching results

Table S7. Saturation indices (*SI*) of selected mineral phases controlling the release and retention of Pb and Zn in the selected SPs.

	$CaSO_4.2H_2O$	$PbSO_4$	FeOOH	$Al(OH)_3$	$Fe(OH)_3$
SPs	Gypsum	Anglesite	Ferrihydride	Gibbsite	Goethite
S1	-2.93	-1.43	4.05	4.76	6.77
S2	-3.45	-2.33	4.58	4.39	7.3
S3	-3.15	-2.19	5.22	4.05	7.94
S7	-2.51	-2.41	5.07	3.61	7.79
S8	-1.55	-2.03	1.85	3.1	4.61

SI > 0: Saturated, SI < 0: Unsaturated.

Table S8. Leachate characteristics of the SPs, including other dissolved minerals.

Sample	T(°C)	ORP (mV)	EC (mS/cm)	Eh (mV)	Alkalinity (meq/L)	Al (mg/L)	Ca (mg/L)	K (mg/l)	SO_4^{2-} (mg/L)	Mg (mg/L)	Fe (mg/L)	Si (mg/L)
S1	22.8	214±30	0.13±0.08	455	0.07	34.6±5.2	9.6±0.62	11.3±1.2	19±1.8	3.6±0.7	16.4±7	63.8±0.6
S2	22.9	177±38	0.06±0.01	418	0.16	32.3±5.5	10.4±1.33	5.8±0.7	5.1±0.7	6.1±1.8	9.46±4	66.9±0.5
S3	22.9	158±57	0.08±0.05	399	0.27	33.8±5.6	13.3±0.4	12.2±1.8	8±0.9	1.9±0.6	18.8±10	62±3.2
S4	22.9	199±27	0.04±0.01	440	0.02	1.3±1.5	2.8±0.21	3.2±0.9	5.4±0.3	0.7±0.3	ND	1.6±0.4
S5	22.9	142±23	0.19±0.02	383	0.56	6.0±5.9	35.4±0.5	5.9±0.6	13±1	2.1±0.6	ND	4.1±0.3
S6	22.2	152±23	0.25±0.03	393	0.32	ND	37.0±0.1	5.7±1.4	20±0.9	5.4±0.1	ND	1.3±0.2
S7	22.8	161±32	0.23±0.01	402	0.29	19.4±2.4	22.8±0.59	14.8±0.6	23±0.3	7.3±1.4	9.6±3	29.1±0.5
S8	23	193±18	0.57±0.05	434	0.22	1.7±3	46.8±1.3	17.7±1.8	166±3.5	29.1±3.4	ND	3.5±0.2

*ND = Not detected.

References

1. USDA,1987.https://www.wcc.nrcs.usda.gov/ftpref/wntsc/H&H/training/soilsother/soil-USDA-textural-class.pdf
